# Supplementary material for: MetaRibo-Seq measures translation in microbiomes
Source: Nat Commun. 2020 Jun 29;11:3268. doi: 10.1038/s41467-020-17081-z (PMC7324362; doi:10.1038/s41467-020-17081-z)
Supplement: Supplementary file 10 — Supplementary Data 7 [file 41467_2020_17081_MOESM10_ESM.zip › File2/Confidence_High_Taxonomy/429816_out.krona.html]

Javascript must be enabled to view this page.

members
magnitude
magnitudeUnassigned
count
unassigned
taxon
rank

429816\_out

132

2
superkingdom
132

132
976
phylum

200643
class
132

171549
order
132

132
815
family

genus
6
816
132

SRS013476\_contig\_number\_17585SRS015431\_contig\_number\_16606SRS019068\_contig\_number\_71960SRS065176\_contig\_number\_6775SRS076929\_contig\_number\_5407SRS1041164\_contig\_number\_1698


SRS058770\_contig\_number\_21876
species
47678
1

species
162156
10

SRS018984\_contig\_number\_6731SRS019030\_contig\_number\_8125SRS019787\_contig\_number\_5332SRS023583\_contig\_number\_2151SRS065176\_contig\_number\_contig-100\_59.69219SRS077127\_contig\_number\_9612SRS140492\_contig\_number\_30145SRS148424\_contig\_number\_27660SRS892882\_contig\_number\_1706SRS893230\_contig\_number\_10752

3
871324
species

SRS020622\_contig\_number\_2944SRS058723\_contig\_number\_90SRS1041145\_contig\_number\_30710


SRS893342\_contig\_number\_1387
1
1263038
species

818
species
1

SRS015890\_contig\_number\_12942

2292944
species
1

SRS062701\_contig\_number\_883

6
820
species

SRS014459\_contig\_number\_33836SRS016897\_contig\_number\_624SRS054905\_contig\_number\_20475SRS142542\_contig\_number\_11209SRS142923\_contig\_number\_21739SRS893342\_contig\_number\_519

species
329854
6

SRS015960\_contig\_number\_1538SRS016954\_contig\_number\_10152SRS024625\_contig\_number\_2923SRS076976\_contig\_number\_contig-100\_1821.122809SRS077194\_contig\_number\_12752SRS148511\_contig\_number\_4738


SRS024435\_contig\_number\_15199
1
291644
species

1
2292002
species

SRS019445\_contig\_number\_33454


SRS024388\_contig\_number\_5093SRS1041038\_contig\_number\_14663SRS1041136\_contig\_number\_11740SRS143523\_contig\_number\_13664SRS148511\_contig\_number\_contig-100\_2736.113753
5
626931
species

2
46506
species

SRS013098\_contig\_number\_36472SRS017859\_contig\_number\_330

83
246787
species

SRS011239\_contig\_number\_11077SRS013800\_contig\_number\_5346SRS013800\_contig\_number\_16300SRS014235\_contig\_number\_26434SRS014287\_contig\_number\_10062SRS015065\_contig\_number\_1614SRS015190\_contig\_number\_2862SRS015190\_contig\_number\_11808SRS015579\_contig\_number\_13349SRS015782\_contig\_number\_18705SRS016018\_contig\_number\_11298SRS016095\_contig\_number\_21023SRS016132\_contig\_number\_400SRS016989\_contig\_number\_9690SRS017191\_contig\_number\_13553SRS017701\_contig\_number\_10258SRS017821\_contig\_number\_5096SRS018427\_contig\_number\_1838SRS018541\_contig\_number\_1329SRS018656\_contig\_number\_12037SRS019161\_contig\_number\_13657SRS019267\_contig\_number\_3845SRS019685\_contig\_number\_25495SRS021948\_contig\_number\_contig-100\_2336.172895SRS024075\_contig\_number\_358SRS024132\_contig\_number\_44789SRS024492\_contig\_number\_10739SRS043411\_contig\_number\_2261SRS043701\_contig\_number\_10287SRS045244\_contig\_number\_6533SRS045713\_contig\_number\_contig-100\_1993.100163SRS047014\_contig\_number\_5826SRS048981\_contig\_number\_5798SRS049446\_contig\_number\_1986SRS049959\_contig\_number\_contig-100\_3821.227645SRS050299\_contig\_number\_9807SRS050801\_contig\_number\_529SRS052697\_contig\_number\_1114SRS053214\_contig\_number\_9264SRS053398\_contig\_number\_23379SRS058070\_contig\_number\_20058SRS058770\_contig\_number\_22142SRS062427\_contig\_number\_6703SRS063040\_contig\_number\_30158SRS063518\_contig\_number\_35401SRS065397\_contig\_number\_11269SRS075341\_contig\_number\_18476SRS075821\_contig\_number\_2236SRS075878\_contig\_number\_13619SRS075984\_contig\_number\_20759SRS077086\_contig\_number\_6866SRS077730\_contig\_number\_3727SRS078419\_contig\_number\_19972SRS097889\_contig\_number\_34SRS097920\_contig\_number\_6731SRS097958\_contig\_number\_8470SRS098073\_contig\_number\_2468SRS098571\_contig\_number\_27012SRS103987\_contig\_number\_2182SRS1041031\_contig\_number\_24592SRS1041132\_contig\_number\_50SRS1041141\_contig\_number\_396SRS1041142\_contig\_number\_contig-100\_3508.46785SRS104400\_contig\_number\_10094SRS104975\_contig\_number\_18158SRS1055034\_contig\_number\_404SRS1055038\_contig\_number\_2739SRS1055083\_contig\_number\_1931SRS140513\_contig\_number\_9549SRS142503\_contig\_number\_20072SRS142923\_contig\_number\_19317SRS143342\_contig\_number\_9383SRS143780\_contig\_number\_contig-100\_1321.158685SRS145497\_contig\_number\_15410SRS146813\_contig\_number\_7729SRS147346\_contig\_number\_1611SRS148784\_contig\_number\_21171SRS148817\_contig\_number\_4255SRS149325\_contig\_number\_11564SRS893172\_contig\_number\_437SRS893187\_contig\_number\_10704SRS893373\_contig\_number\_12754SRS971275\_contig\_number\_32071


SRS053649\_contig\_number\_600
species
821
1

1
371601
species

SRS893187\_contig\_number\_12960

1
species
626929

SRS077335\_contig\_number\_3092

species
1262753
1

SRS1041092\_contig\_number\_11204

species
28111
1

SRS020233\_contig\_number\_53946
